# Socioeconomic deprivation as a determinant of cancer mortality and the Hispanic paradox in Texas, USA

**DOI:** 10.1186/1475-9276-12-26

**Published:** 2013-04-15

**Authors:** Billy U Philips Jr, Eric Belasco, Kyriakos S Markides, Gordon Gong

**Affiliations:** 1 F. Marie Hall Institute for Rural and Community Health, Texas Tech University Health Sciences Center, Lubbock, TX, 79430, USA; 2 Department of Family and Community Medicine, Texas Tech University Health Sciences Center, Lubbock, TX, 79430, USA; 3 Department of Agricultural Economics and Economics, Montana State University, Bozeman, MT, 59717-2920, USA; 4 Division of Sociomedical Sciences in Preventive Medicine and Community Health, University of Texas Medical Branch, Galveston, TX, 77555, USA

## Abstract

**Introduction:**

We have recently reported that delayed cancer detection is associated with the Wellbeing Index (WI) for socioeconomic deprivation, lack of health insurance, physician shortage, and Hispanic ethnicity. The current study investigates whether these factors are determinants of cancer mortality in Texas, the United States of America (USA).

**Methods:**

Data for breast, colorectal, female genital system, lung, prostate, and all-type cancers are obtained from the Texas Cancer Registry. A weighted regression model for non-Hispanic whites, Hispanics, and African Americans is used with age-adjusted mortality (2004–2008 data combined) for each county as the dependent variable while independent variables include WI, percentage of the uninsured, and physician supply.

**Results:**

Higher mortality for breast, female genital system, lung, and all-type cancers is associated with higher WI among non-Hispanic whites and/or African Americans but with lower WI in Hispanics after adjusting for physician supply and percentage of the uninsured. Mortality for all the cancers studied is in the following order from high to low: African Americans, non-Hispanic whites, and Hispanics. Lung cancer mortality is particularly low in Hispanics, which is only 35% of African Americans’ mortality and 40% of non-Hispanic whites’ mortality.

**Conclusions:**

Higher degree of socioeconomic deprivation is associated with higher mortality of several cancers among non-Hispanic whites and African Americans, but with lower mortality among Hispanics in Texas. Also, mortality rates of all these cancers studied are the lowest in Hispanics. Further investigations are needed to better understand the mechanisms of the Hispanic Paradox.

## Introduction

A steady decline in cancer mortality in the United States has been observed in the past ten years, which is attributed to the reduction in the number of smokers, increased cancer screening, and better treatment [1,2]. However, disparities in cancer mortality persist due to socioeconomic inequality, among other factors. For example, a lack of health insurance among poorer people limits their access to regular primary care in general and to cancer screening in particular, which may result in delayed cancer detection and treatment as well as increased mortality rates. Philips et al. [3] recently reported the results of principle component analysis (PCA) that a higher percentage of people with lung-bronchial, female genital system, and all-type cancers of advanced stages at first diagnosis among Texas counties were associated with a higher degree of socioeconomic deprivation as measured by the Wellbeing Index (WI) originally developed by Albrecht and Ramasubramanian [4]. Further studies by Belasco et al. [5] have shown that delayed detection of breast, colorectal, and lung-bronchial cancers are associated with the Health Care Accessibility Index, which is the first principal component score of a PCA when physician supply (the ratio of the number of physicians to population served) and percentage of residents without health insurance in a given county are combined with variables contained in the WI. The Philips team further demonstrated that Hispanics in Texas had a higher percentage of people with late-stage cancers (breast, lung, colorectal, female genital system, and prostate) at initial diagnosis in a univariate analysis in the study focused on county level population data [6]. This result is consistent in principle with findings by others that Hispanics tend to have a higher rate of late-stage cancers for lung, colorectal, prostate, and melanoma of the skin based on data from the 17 registries of the United States in Surveillance, Epidemiology, and End Results (SEER) Program [7]. However, after controlling for socioeconomic status, physician supply, and the percentage of the uninsured, we found that the higher percentage of Hispanics was associated with a lower percentage of late-stage lung and colorectal cancer rate at diagnosis [6], a novel confirmation of the Hispanic Paradox [8]. The present study further investigates whether socioeconomic deprivation as measured by the WI, lack of health insurance, and physician supply are determinants of cancer mortality in Hispanics, non-Hispanic whites, and African Americans in Texas, USA. We also determine whether the Hispanic Paradox in cancer mortality is present in the context of socioeconomic deprivation.

## Methods

### Sources of data

Cancer mortality data from 2004 to 2008 were provided by the Texas Cancer Registry, Cancer Epidemiology and Surveillance Branch, the Texas Department of State Health Services (data available up to 2008) [9]. This database provides cancer data by year, age, county, ethnicity (Hispanic vs. Non-Hispanic), race, as well as population size for each county; the large span of five years offers more stable rates in the population, particularly in counties with a small population size. Three racial/ethnic groups are studied including Hispanics, non-Hispanic whites, and non-Hispanic African Americans. Cancer data between 2004 and 2008 are combined (the latest available data is the 2008 data) to match the five years of 2005–2009 American Community Survey data [10] (with a one-year lap) with “county” as a unit of observation. Cancer categories studied include breast, colorectal, female genital system, lung-bronchial, prostate, and all-type cancers. Female genital system includes cervix uteri, ovary, corpus and uterus, vagina, vulva, and others. We pool the five-year data to calculate age-adjusted mortality (deaths per 100,000 people) using the 2000 USA standard population [11].

Socioeconomic variables used to construct the Wellbeing Index (WI) are derived from the 2005–2009 American Community Survey (US Census Bureau) [10]. Since this survey does not currently provide the percentage of people with disabilities, the remaining nine of the ten socioeconomic variables originally used to build the WI were employed in this model. These socioeconomic variables include public income support, homeownership, bedroom overcrowding, educational attainment, single parental household, poverty status, vehicle ownership, unemployment, and home telephone service. The first principal component scores of the 254 counties are used as a continuous variable for WI with larger values indicating a higher degree of socioeconomic deprivation. The first principle component scores are standardized with a mean of 0, and one standard deviation as 1. The reason why we use WI is to gain the advantage of a uniform index for a coherent socioeconomic profile and to avoid multicollinearity and a potentially inflated variance if used unchecked in the multiple regression. PCA is a variable-reduction technique, producing a single composite index to represent multiple correlated variables.

Data for the percentage of uninsured residents in each county are obtained from Texas State Data Center [12]. The number of physicians and estimated population size in each county from 2004 to 2008 are derived from Texas Department of State Health Services (DSHS) [13]. Physician supply is the number of physicians per 1,000 residents in each county. Physicians considered are those with medical doctor (MD) and/or doctor of osteopathy (DO) degrees who worked directly with patients. Residents and fellows, teachers, administrators, researchers, and those who were working for the federal government, military, retired, or not in practice, were excluded from the total of physicians by DSHS [13].

### Statistical analysis

A Poisson model was performed with the GENMOD procedure (SAS, Cary, NC) with cancer mortality as the response variable, while explanatory variables included WI, age (0–44, 45–54, 55–64, 65–74, 75–84 and ≥ 85 years), sex, ethnicity, physician supply, and percentage of the uninsured in a county. Weighted linear multiple regression was also performed with the GLM procedure with population size as the weight variable. The response variable is age-adjusted cancer mortality and the explanatory variables are WI, sex, physician supply, and percentage of the uninsured in a county analyzed separately for non-Hispanic whites, African Americans and Hispanics. Weighted Tobit regression model (with the SAS QLIM procedure) is also performed where cancer mortality at 0 is censored for counties without a single cancer death within a particular category of cancer in the five-year interval. This tends to produce a conservative estimate of the underlying theoretical mortality rate in the population segments. We also estimated age-specific mortality rates among counties with different socioeconomic status (five categorical WIs, WI_1_ to WI_5,_ from low to high degree of socioeconomic deprivation) according to the method by Soto-Salgado et al. [14]. In addition, we estimated age-standardized mortality and the standardized rate ratio in counties with WI_1_ vs. WI_5_ according to Torres-Cintrón, et al. [15].

This study was approved by Texas Tech University Health Sciences Center Institutional Review Board with expedited review because of its use of anonymous public source and published data. The authors declare no conflicts of interests regarding this investigation.

## Results

### Mortality from the Poisson model

Table 1 shows that mortality of all categories of cancer is positively associated with WI with the exception of prostate cancer mortality, which is negatively associated with WI. Mortality is significantly lower in females vs. males in all categories of cancer involving both sexes. Mortality in each age group is significantly higher than the next younger age group in all categories of cancer with a few exceptions. Compared with non-Hispanic whites, African Americans had significantly higher mortality rates for all-type, colorectal, and lung-bronchial cancers, but had significantly lower mortality rates for prostate, breast, and female genital system cancers after adjusting for confounders. Hispanics had the lowest mortality rate for all the six categories of cancer studied.

**Table 1:** Results of Poisson regression analysis of cancer mortality in Texas

	**All-Type**				**Prostate**			
**Parameter**	**Estimate**	**95% C.I.**		***p***	**Estimate**	**95% C.I.**		***p***
WI	0.03	0.03	0.03	<.0001	−0.02	−0.03	−0.02	<.0001
F vs. M	−0.37	−0.37	−0.37	<.0001				
Age (y) 0-44	−5.02	−5.03	−5.01	<.0001	0.95	0.91	0.98	<.0001
45-54	−2.64	−2.65	−2.63	<.0001	−0.05	−0.11	0.01	0.0866
55-64	−1.63	−1.64	−1.63	<.0001	−10.50	−10.72	−10.27	<.0001
65-74	−0.83	−0.83	−0.82	<.0001	−5.71	−5.76	−5.65	<.0001
75-84	−0.28	−0.29	−0.28	<.0001	−3.69	−3.72	−3.66	<.0001
African Am	0.38	0.38	0.39	<.0001	−2.17	−2.19	−2.15	<.0001
Hispanic	−0.23	−0.24	−0.22	<.0001	−1.04	−1.06	−1.02	<.0001
% Uninsured	−0.01	−0.01	−0.01	<.0001	−0.01	−0.01	0.00	<.0001
Physician	−0.03	−0.03	−0.03	<.0001	−0.03	−0.04	−0.01	<.0001
	Colorectal				Lung-Bronchial			
WI	0.01	0.01	0.02	0.0004	0.04	0.03	0.04	<.0001
F vs. M	−0.44	−0.45	−0.43	<.0001	−0.55	−0.56	−0.54	<.0001
Age (y) 0-44	−5.53	−5.56	−5.50	<.0001	−5.75	−5.78	−5.73	<.0001
45-54	−2.95	−2.97	−2.93	<.0001	−2.50	−2.52	−2.49	<.0001
55-64	−1.97	−1.99	−1.96	<.0001	−1.23	−1.25	−1.22	<.0001
65-74	−1.26	−1.27	−1.24	<.0001	−0.26	−0.27	−0.25	<.0001
75-84	−0.56	−0.58	−0.55	<.0001	0.15	0.14	0.16	<.0001
African Am	0.62	0.60	0.64	<.0001	0.18	0.16	0.20	<.0001
Hispanic	−0.13	−0.17	−0.09	<.0001	−1.08	−1.10	−1.05	<.0001
% Uninsured	−0.01	−0.01	0.00	<.0001	0.00	0.00	0.00	<.0001
Physician	−0.08	−0.09	−0.07	<.0001	−0.06	−0.06	−0.06	<.0001
	Breast				Female Genital System			
WI	0.03	0.03	0.04	<.0001	0.04	0.03	0.05	<.0001
Age (y) 0-44	0.54	0.52	0.57	<.0001	0.40	0.36	0.43	<.0001
45-54	−0.18	−0.22	−0.14	<.0001	0.13	0.08	0.18	<.0001
55-64	−3.99	−4.01	−3.96	<.0001	−3.86	−3.89	−3.83	<.0001
65-74	−1.72	−1.74	−1.70	<.0001	−1.85	−1.88	−1.83	<.0001
75-84	−1.09	−1.11	−1.07	<.0001	−1.11	−1.13	−1.08	<.0001
African Am	−0.75	−0.77	−0.73	<.0001	−0.50	−0.53	−0.48	<.0001
Hispanic	−0.36	−0.38	−0.34	<.0001	−0.11	−0.13	−0.08	<.0001
% Uninsured	−0.01	−0.01	−0.01	<.0001	−0.01	−0.01	−0.01	<.0001
Physician	0.01	0.00	0.02	0.1308	0.02	0.01	0.03	0.0004

Note: All age groups are compared with the group of 85 years or older. Y = years; Am = American; African American and Hispanic are compared with non-Hispanic whites. F = Female; M = Male.

### Age-adjusted mortality in relation to ethnicity

**Figure 1:**
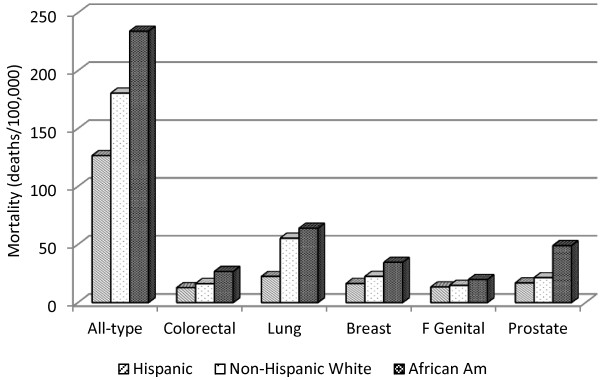
Cancer mortality among different ethnic groups in Texas (African Am: African American).

When cancer cases in the entire State of Texas are combined by ethnicity, age-adjusted mortality of all six categories of cancer studied are in the following order from high to low: African Americans, non-Hispanic whites, and Hispanics (Figure 1). The ethnic difference in mortality is notably larger for lung cancer: mortality rate in Hispanics is only 35% of African Americans’ mortality and 40% of non-Hispanic whites’ mortality. On the other hand, prostate cancer mortality in African Americans is several times that of non-Hispanic whites or Hispanics.

### Age-adjusted mortality in relation to WI

Weighted multiple regression analysis with GLM shows that age-adjusted mortality for all-type, breast, female genital system and lung cancers is positively correlated with WI among African Americans while mortality of all-type, colorectal, and lung cancers is positively and significantly correlated with WI among non-Hispanic whites (Table 2). Similar results are obtained with the QLIM Tobit model. Paradoxically, mortality of all-type, breast, female genital system, and lung cancers is negatively correlated with WI in Hispanics (Table 2), although its colorectal cancer mortality is positively associated with WI.

**Table 2:** Results of weighted multiple regression analysis of cancer mortality in Texas

	**Non-Hispanic White**				**African American**				**Hispanic**			
**Cancer Param.**	**Est.**	**95% C.I.**		***p***	**Est.**	**95% C.I.**		***p***	**Est.**	**95% C.I.**		***p***
All-type												
WI	5.9	3.3	8.5	<.0001	18.0	10.8	25.2	<.0001	−39	−62	−15	0.0014
Sex (F vs. M)	−72	−76	−68	<.0001	−132	−141	−123	<.0001	−49	−92	−6	0.0268
% Uninsured	3.6	2.4	4.7	<.0001	−1.3	−2.6	0.0	0.0486	2.1	−1.4	5.6	0.2384
Physician	−9.8	−13	7.0	<.0001	5.07	−3.2	13.3	0.2266	8.0	−28	44	0.6681
Breast												
WI	0.32	−0.4	1	0.3535	5.2	3.1	7.4	0.0001	−4.8	−8.2	−1.3	0.0073
% Uninsured	-.04	−0.3	0.3	0.8054	−0.2	−0.5	0.2	0.3535	−1.3	−1.9	−0.8	0.0001
Physician	−0.1	-.90	.68	0.777	−1.2	−3.6	1.3	0.3361	12.2	6.8	17.6	0.0001
Colorectal												
WI	0.7	0.3	1.1	0.0019	1.0	−0.7	2.7	0.2564	12	3	21	0.0064
Sex (F vs. M)	−6.6	−7.3	−6.0	<.0001	−12	−15	−10	<.0001	−1	−17	15	0.8904
% Uninsured	0.4	0.2	0.6	<.0001	0.1	−0.2	0.4	0.4908	0.45	-.82	1.71	0.4862
Physician	−1.9	−2.5	−1.4	<.0001	0.32	−1.6	2.26	0.7474	−1.4	−15	11.9	0.8341
F. Genital												
WI	0.5	−0.1	1	0.0799	3	1.1	4.9	0.0024	−18	−26	−10	0.0001
% Uninsured	.02	−0.2	0.3	0.8850	−0.3	−0.6	0.1	0.1324	0.1	−1.1	1.3	0.8687
Physician	0.2	0.45	0.84	0.5564	−1.5	−3.7	0.76	0.196	−9.4	−22	3.1	0.1408
Lung												
WI	2.2	0.9	3.5	0.0012	10.7	7.3	14.2	<.0001	−10	−16	−3	0.0025
Sex (F vs. M)	−27	−29	−25	<.0001	−57	−62	−53	<.0001	−29	−41	−17	<.0001
% Uninsured	1.8	1.2	2.3	<.0001	−0.5	−1.2	0.1	0.0866	1.98	1.05	2.91	<.0001
Physician	−4. 5	−6.1	3.0	<.0001	-.71	−4.7	3. 3	0.7259	3.03	−6.8	12.8	0.5426
Prostate												
WI	−0.5	−1.3	0.3	0.1851	−1.1	−5.3	3.2	0.6236	−7.4	−21	5.9	0.274
% Uninsured	0.3	0	0.7	0.0810	−0.3	−1.1	0.5	0.4708	1	−0.9	3	0.2989
Physician	−1.1	−2.0	−0.1	0.0279	1.9	−2.9	6.8	0.4302	−11.7	−32	8.7	0.2598

### Age-adjusted mortality in relation to percent uninsured

Hispanics had the highest percentages of uninsured, followed by non-Hispanic Black-Other ethnic group, and Anglos had the lowest. Age-adjusted mortality of all-type, colorectal, and lung cancer is positively correlated with the percentage of uninsured individuals among non-Hispanic whites. Among African Americans, age-adjusted mortality is not associated with the percentage of uninsured individuals for all categories of cancer except for all-type cancer, which is negatively associated with the percentage. Age-adjusted mortality for lung-bronchial cancer is positively associated with the percentage of the uninsured, while age-adjusted breast cancer mortality is negatively correlated with the percentage among Hispanics (*p* = 0.0001, Table 2).

### Age-adjusted mortality in relation to physician supply

As expected, mortality of certain categories of cancer (all-type, colorectal, and lung) is negatively correlated with physician supply among non-Hispanic whites (Table 1). However, mortality for any cancers studied is not correlated with physician supply among African Americans, while breast cancer mortality is paradoxically positively correlated with physician supply in Hispanics (Table 2).

### Age-specific and age-standardized mortality among categorical WI

Table 3 shows that age-specific mortality varies according to categorical WI and cancer type. In many cases, mortality rate of WI_5_ is the lowest, while mortality rate of WI_1_ is significantly higher than that of WI_5_ for many age groups for several cancers as judged by their ratio and confidence interval at α = 0.05; after Bonferroni correction for multiple comparisons, most of the differences are not statistically significant. Table 4 shows that age-standardized mortality in WI_5_ is the lowest in most cancers and is significantly lower than that of WI_1_.

**Table 3:** Age-specific cancer mortality by categorical WI

**Cancer**	**WI**_**Category**_	**45-54 yrs**	**55-64 yrs**	**65-74 yrs**	**75-84 yrs**	**≥85 yrs**	**Overall**
All	1	98	296	680	1,250	1,599	393.7
	2	119	339	757	1,252	1,580	469.6
	3	124	344	753	1,268	1,569	494.3
	4	124	331	726	1,219	1,568	447.9
	5	108	277	620	1,003	1,355	418.1
	WI_1_/WI_5_	0.91	1.07	1.10	1.25	1.18	0.94
	95% C.I.	0.85-0.97	1.03-1.11	1.06-1.13	1.21-1.28	1.13-1.23	0.92-1.23
Colorectal	1	10	27	57	115	202	37.4
	2	13	30	58	122	191	44.3
	3	12	32	60	125	195	47.4
	4	11	30	63	115	195	42.8
	5	10	27	53	99	157	40.3
	WI_1_/WI_5_	0.99	1.01	1.09	1.16	1.29	0.93
	95% C.I.	0.81-1.17	0.87-1.14	0.96-1.21	1.05-1.27	1.15-1.43	0.87-0.99
Lung	1	22	90	245	388	307	117.4
	2	28	106	281	401	317	144.4
	3	32	104	282	399	307	151.2
	4	27	95	246	363	304	126.9
	5	16	67	181	263	239	101.7
	WI_1_/WI_5_	1.33	1.36	1.35	1.48	1.28	1.15
	95% C.I.	1.19-1.46	1.27-1.44	1.291.41	1.41-1.54	1.17-1.40	1.12-1.19
Breast	1	22	53	76	117	179	55.2
	2	31	52	72	101	156	58.8
	3	29	56	71	112	149	61.3
	4	30	57	81	120	162	62.9
	5	30	44	65	79	136	52.8
	WI_1_/WI_5_	0.74	1.20	1.18	1.48	1.32	1.05
	95% C.I.	0.59-0.90	1.051.34	1.03-1.32	1.33-1.63	1.13-1.50	0.98-1.11
Female	1	12	28	53	89	106	34.4
	2	13	29	59	77	83	37.0
	3	15	32	51	91	96	40.6
	4	17	33	61	89	95	40.8
	5	18	32	55	75	92	40.5
	WI_1_/WI_5_	0.68	0.86	0.97	1.19	1.15	0.85
	95% C.I.	0.47-0.89	0.68-1.04	0.80-1.13	1.03-1.36	0.92-1.38	0.77-0.93
Prostate	1	2	14	59	202	517	39.8
	2	2	14	67	204	569	49.9
	3	3	12	67	185	564	51.4
	4	2	15	65	204	543	46.8
	5	3	11	62	161	477	50.2
	WI_1_/WI_5_	0.65	1.29	0.94	1.26	1.08	0.79
	95% C.I.	0.12-1.19	1.00-1.58	0.78-1.11	1.13-1.39	0.94-1.23	0.71-0.87

**Table 4:** Age-standardized mortality (deaths/100,000) by categorical WI

	**WI**_**1**_	**WI**_**2**_	**WI**_**3**_	**WI**_**4**_	**WI**_**5**_	**W**_**1**_**/W**_**5 **_**(95% C.I)**
Women						
All	146.1	154.0	151.1	149.8	126.2	1.16 (1.13-1.19)
Colorectal	13.6	14.6	14.3	13.7	11.3	1.20 (1.10-1.30)
Lung	40.4	42.5	41.8	37.2	24.2	1.67 (1.58-1.76)
Breast	22.4	22.5	22.6	24.5	20.0	1.12 (1.05-1.20)
F. Genital	14.3	14.5	15.4	16.1	15.5	0.92 (0.86-1.00)
Men						
All	210.2	227.7	235.2	226.3	192.1	1.09 (1.07-1.12)
Colorectal	19.9	20.6	21.9	21.8	18.8	1.05 (0.98-1.13)
Lung	63.4	73.0	74.2	68.0	51.9	1.22 (1.18-1.27)
Prostate	22.4	22.5	22.6	24.5	20.0	1.12 (1.05-1.20)

## Discussion

This study, for the first time, reports that age-adjusted mortality rates of all-type, lung and/or female genital system cancers are positively and significantly correlated with socioeconomic deprivation as measured by the WI among non-Hispanic whites and African Americans, but paradoxically negatively correlated with WI among Hispanics. These results in non-Hispanic whites and/or African Americans (but not Hispanics) resonate with the results of the previous study showing that the percentage of late-stage cases (a measure of delayed diagnosis) of all-type, lung, and female genital system cancers is positively and significantly correlated with WI in Texas counties [3]. Thus, the association between WI and delay in the diagnosis of these cancers coincide with the association between WI and age-adjusted mortality of the same cancers in non-Hispanic whites and/or African Americans. This makes it plausible that a causal relationship may exist between delayed diagnosis and increased mortality rates of these cancers.

Moreover, this study shows that age-adjusted mortality of colorectal cancer is also positively correlated with WI among non-Hispanic whites, although the ratio of late-to-early stage cases of colorectal cancer is not correlated with WI in the earlier report [3]. As discussed earlier, the lack of correlation between WI and delayed colorectal cancer diagnosis is partly due to polypectomy more frequently seen in socioeconomically better-off counties, which reduces cancer cases at the early stage [3]. On the other hand, polypectomy tends to reduce the risk of colorectal cancer and hence its mortality, resulting in a positive correlation between WI and colorectal cancer mortality. Lack of insurance will lead to a higher percentage of late-stage colorectal cancer cases and higher mortality in all ethnic groups including Hispanics.

We emphasize that the positive association between age-adjusted colorectal cancer mortality and WI is not observed in African Americans, signifying the ethnic difference. Conversely, a positive association between age-adjusted breast cancer mortality and WI is observed in African Americans but not in non-Hispanic whites. The absence of association between age-adjusted breast cancer mortality and WI in non-Hispanic whites may be due to the fact that breast cancer incidence is very high among this ethnic group (due to genetic predisposition to breast cancer which is hardly modified by environmental factors such as socioeconomic deprivation) in addition to the lack of effective measures for early detection and treatment regardless of health insurance or socioeconomic status. Such a phenomenon is termed “length-time bias” because more aggressive tumors such as breast cancer often develop into advanced-stages before the next screening [16].

One of the unexpected results is the finding that higher age-adjusted mortality in four of the six categories of cancer studied is paradoxically associated with lower degree of socioeconomic deprivation (i.e., lower WI) in Hispanics, in sharp contrast with findings in non-Hispanic whites and African Americans. To the best of our knowledge, this is the first report of such a unique “Hispanic Paradox”. The paradox may be due to the following facts. The WI map [3] is very similar to the map of the percentage of Hispanic population [17], especially along the Mexico-US border. Thus, the WI to a certain degree reflects the percentage of Hispanics (i.e., they are correlated) who tend to have lower mortality in all categories of cancer studied (Figure 1), resulting in a paradoxical association of higher WI with lower mortality. The influences of WI and Hispanic ethnicity on the age-adjusted mortality of certain cancers are in opposite directions. When both Hispanic ethnicity and socioeconomic deprivation are at play, Hispanic ethnicity predominates over socioeconomic deprivation in their effect on the mortalities of certain cancers. The correlation between WI and percentage of Hispanics points to an inherent shortcoming of the WI as a measure of socioeconomic deprivation in predicting health status (a weaker predictor than Hispanic ethnicity in certain cases). Also, the WI may reflect the socioeconomic status of the predominant ethnic group (e.g., Hispanics along the Mexico border and non-Hispanic whites in non-border counties). The findings that age-standardized mortality and age-specific mortality is higher in category WI_1_ than WI_5_ in certain age groups and cancer types may similarly reflect the fact that more Hispanics reside in counties with WI_5_. Clearly, it is inadequate to predict cancer mortality solely based on socioeconomic status; ethnic differences must be taken into consideration. An exception is that higher colorectal cancer mortality is positively associated with higher WI in Hispanics, which may reflect the fact that colonoscopy plays a very critical role in cancer control; colonoscopy is more expensive than screening of other cancers and therefore is more dependent on socioeconomic status in Hispanics as well.

Multiple regression with the Poisson model has provided an overall assessment when all dependent variables, including age and ethnicity, are entered as covariates where mortality is not standardized by age with a standard population. Specifically, the results show that mortality is increasing with age for all categories of cancer with a few exceptions for certain cancers. Compared with non-Hispanic whites, mortality for any category of cancer studies is lower in Hispanics, consistent with the results of analysis where mortality is age-adjusted with a standard population (Figure 1). However, the Poisson model shows that mortality rates for several cancers (prostate, breast, and female genital system) are significantly lower in African Americans than non-Hispanic whites, in “contradiction” with their respective age-adjusted mortality rates seen in Figure 1. In fact, this may not be a contradiction because the results are obtained from different types of analysis: mortality is age-adjusted with a standard population for the latter (Figure 1), but is “adjusted” for all variables (the percentage of the uninsured, physician supply, etc.) in the former (Poisson model). In other words, if health insurance coverage, physician supply, and other conditions were similar (kept constant), African Americans should have had lower rates for these cancers. In addition, this “contradiction” could be partly due to the fact that in the Poisson model, mortality was not age-adjusted with a standard population, which is a common practice for comparison among different populations. Rather, age is entered as a covariate in the Poisson model. Cautions should be taken when mortality is not age-standardized when comparing different populations.

Currently, we do not know the exact mechanisms whereby Hispanics tend to have much lower age-adjusted mortality rate compared with non-Hispanic whites and African Americans. Most accepted is a theory of the selective immigration of healthy immigrants [8]. We hypothesize that the difference in cancer mortality between Hispanics and non-Hispanic whites is largely attributed to the difference in the percentage of smokers. The U.S. Center for Disease Control and Prevention reported that the percentage of smokers were much lower among Hispanics than non-Hispanic white or African Americans [18]. Also, the American Cancer Society recently reported that tobacco use accounts for at least 30% of all cancer deaths and 87% of lung cancer deaths [19]. If this ethnic difference in cancer mortality is related to the difference in percentage of smokers, then the following features should be observed: (1) The difference in lung cancer mortality should be larger than that of all cancer mortality among populations with different percentages of smokers. (2) After adjustment for the percentage of smokers, differences in both mortality rate for lung cancer and all cancer should be reduced. We calculated mortality rate among non-Hispanic whites when assuming the percentage of smokers is the same as that in Hispanics (14.4%) [19]. The results showed that the mortality in non-Hispanic whites is very close to that of Hispanics for both lung cancer mortality and all cancer mortality. Similarly, when the percentage of smokers in Hispanics is the same as non-Hispanic whites (24.2%) [19] both lung cancer mortality and all cancer mortality are almost the same as that of non-Hispanic whites. This Fewer Smokers Hypothesis for the Hispanic Paradox in cancer mortality was first presented by Philips et al. in early 2011 [20]. Blue and Fenelon have recently demonstrated that tobacco usage explains more than 50% of the difference in life expectancy [21]. These findings emphasize the importance of smoking cessation for effective cancer control.

### Limitations

There are several limitations in this study. One of the limitations of analyzing aggregate data such as ours is that results found in ethnic groups, counties, etc. may not apply to every member of the ethnic group, county, etc. Cautions should be taken when interpreting the results. Also, many cancer cases with unknown race or unknown Hispanic origin (1.6%) are excluded from data analysis. However, we believe that this small percentage would not significantly bias our results. This notion is supported by the fact that The Texas Cancer Registry meets the nation’s high quality data standards [9].

## Conclusions

Higher degree of socioeconomic deprivation is associated with higher mortality rates of several cancers among non-Hispanic whites and/or African Americans and is paradoxically associated with lower mortality of most cancers studied among Hispanics. This Hispanic Paradox may result from the fact that the percentage of Hispanics is correlated with the WI. The fact that the ethnic difference in lung cancer mortality is much larger than that of all cancer mortality supports the recently proposed Fewer-Smoker Hypothesis of the Hispanic Paradox. Tobacco-smoking cessation is important for effective cancer control.

## Abbreviations

WI: Wellbeing index; DSHS: Texas Department of State Health Services; USA: United States of America.

## Competing interests

All authors declare no competing interests.

## Authors’ contributions

All the authors contributed to this research. BUP and GG conceived the idea of association between socioeconomic deprivation and cancer mortality among ethnic groups. EB and GG are responsible for study design and statistical analysis. KSM participated in development of the manuscript including literature search/review, editing, and discussion particularly in the Hispanic Paradox. All authors read and approved the final manuscript.

## Authors’ information

BUP: Executive Vice President of Texas Tech University Health Sciences Center and Director of F. Marie Hall Institute for Rural & Community Health. Epidemiologist.

GG: Biostatistician.

EB: Biostatistician, Assistant Professor of Economics, Expert in Health Economics.

KSM: Professor of Aging Studies and Director of Division of Sociomedical Sciences, Dept. of Preventive Medicine and Community Health.
